# Integrating modern and herbal medicines in controlling malaria: experiences of orthodox healthcare providers in Ghana

**DOI:** 10.1186/s13690-024-01472-5

**Published:** 2024-12-23

**Authors:** Irene G. Ampomah, Genevieve A. Ampomah, Theophilus I. Emeto

**Affiliations:** 1 Department of Population and Health, University of Cape Coast, Cape Coast, UC 182, Ghana; 2 Public Health and Tropical Medicine, James Cook University, Townsville, Queensland 4811 Australia; 3 Department of Sociology, Kwame Nkrumah University of Science and Technology, Kumasi, Ghana; 4 World Health Organization Collaborating Centre for Vector-Borne and Neglected Tropical Diseases, James Cook University, Townsville, Queensland 4811 Australia

**Keywords:** Healthcare providers, Herbal medicine, Malaria, Public health, System integration

## Abstract

**Background:**

In Ghana, the government has integrated herbal medicine into the formal healthcare system in response to widespread use of traditional remedies. However, empirical evidence supporting the contribution of integrated healthcare to malaria control remains limited. This study employed a phenomenological qualitative research design to explore the experiences of medical doctors and pharmacists from the coastal, forest and savannah regions of Ghana regarding the integration of modern and herbal medicine in the treatment and control of malaria. Donabedian’s framework for evaluating the quality of healthcare served as the foundational theoretical framework for this research.

**Methods:**

Data were collected through individual in-depth interviews involving 26 participants and analysed using a framework analytical approach.

**Results:**

The findings revealed that inadequate political commitment to the practice of integration has led to several challenges, including the high cost of herbal anti-malaria medications, limited promotional activities surrounding integration, a shortage of qualified medical herbalists, inconsistent supply chains for herbal anti-malaria treatments, and a lack of standardisation in herbal medicine practices. Participants had divergent views regarding the impact of integration on malaria control; while medical doctors believed that the intervention has not significantly contributed to reducing malaria prevalence in Ghana, pharmacists viewed the presence of herbal clinics within government hospitals as an effective and sustainable alternative for treating malaria.

**Conclusion:**

Reflecting on these results, it is imperative for policymakers to explore strategies that could enhance the effectiveness of an integrated health system, thereby increasing the contribution of herbal medicine towards achieving a malaria free nation. Future research could benefit from including policymakers, heads of health directorates, and community members, regarding the role of public health interventions in addressing health inequities in Ghana.

**Supplementary Information:**

The online version contains supplementary material available at 10.1186/s13690-024-01472-5.

**Table:** 

**Text box 1. Contributions to the literature**
• Ghana has been practicing integrated healthcare for more than a decade. However, studies supporting the role of integration in the management of infectious diseases, specifically malaria, are limited.
• Institutional impediments continue to undermine the effectiveness of herbal medicine integration in Ghana.
• Ghanaian pharmacists perceive the merging of the two systems as a means of preserving the potency of conventional anti-malaria medications. Medical doctors, however, disagree with this assertion.
• Achieving effective malaria management through integration calls for critical review of health policies to eliminate integration barriers and bring all relevant stakeholders on board.

## Introduction

Herbal medicine encompasses traditional practices for managing, treating, and maintaining health that predate the establishment of modern biomedical healthcare systems [1]. In this research, herbal medicine refers to the use of medicinal plants for healing purposes [2]. Anthropologic and cross-cultural studies suggest that familiar disease episodes such as cuts, foot rots among others [3] are often treated outside formal health systems [4]. Globally, a variety of herbs, plant extracts, mineral substances, and animal products have been utilised for health promotion and disease prevention [5].

In many regions of Africa, the use of herbal medicine is culturally accepted and prevalent, serving as a common treatment for a diverse array of ailments [6, 7]. In Ghana, herbal medicine is widely recognised and utilised, with evidence suggesting a growing trend in its adoption [8]. Ghanaians frequently turn to herbal remedies for the treatment and management of a broad spectrum of ailments, ranging from acute to severe conditions [3]. Research conducted among Ugandan and Ghanaian expectant mothers seeking antenatal care has demonstrated the use of herbal medicines to alleviate abdominal pain, constipation, and to promote pregnancy protection and a smooth delivery [9, 10].

Malaria remains a significant public health burden in Ghana, accounting for over 30% of outpatient visits and 23% of inpatient admissions [11]. The country experiences diverse transmission patterns, with intermittent transmission in the capital, heavy seasonal transmission in the Upper West Region, and seasonal transmission in other areas [11]. Herbal remedies are widely used in Ghana to treat malaria and associated fevers [12]. This practice is not unique to Ghana, as it has been observed in other African countries. For example, in Côte d’Ivoire, *Warburgia salutaris (Pycnanthus angolensis)* and *Nauclea latifolia* have been identified as effective treatments for malaria [13, 14]. In Ghana, a combination of modern and herbal medicine is often utilised by malaria patients [12].

Ghanaians sometimes use herbal medicine alone to treat malaria, while other times it is used to complement orthodox anti-malarial therapies [5, 15, 16]. For example, nibima a medicinal plant also known as *Cryptolepis sanguinolenta* is a commonly used and scientifically proven treatment for malaria [17]. Tea formulation derived from nibima (Phyto-Laria) has been reported to achieve a 93.5% cure rate without adverse side effects [17]. Other herbal remedies with potential anti-plasmodial activity against malaria in Ghana include *Tridax procumbens*,* Phyllanthus amarus*,* Theobroma cacao*,* Haematostaphis barteri*,* and Plumeria alba* [15].

Several factors contribute to the preference for herbal remedies over modern medicine among those suffering from malaria. Such factors include perceived effectiveness, minimal adverse effects, and geographical and financial accessibility [6, 8, 9, 12, 18]. Hence, health service users often go to great lengths to obtain herbal medicines. However, the widely held belief that herbal remedies are both safe and cost-effective is contentious, as some studies present contrary findings [19–24].

To direct Ghanaians towards competent medical herbalists and optimise the benefits associated with herbal medicine have led to efforts to integrate herbal practices into the modern healthcare system. This intervention was initiated through policy reforms and the establishment of regulatory bodies [25]. The 2005 Herbal Medicine Practice Policy and the subsequent formation of the Traditional Medicine Practice Council (TMPC) in 2010 laid the groundwork for this integration. The publication of the Ghana Herbal Pharmacopoeia in 2007 further solidified the recognition and standardisation of herbal medicines. By 2012, herbal medicine was formally integrated into the primary healthcare system, with initial implementation in 18 public hospitals [5]. 

This integration has enabled formally trained medical herbalists from institutions such as the Kwame Nkrumah University of Science and Technology (KNUST) and the Centre for Scientific Research into Plant Medicine (CSRPM) to practice alongside conventional healthcare providers. While the integration of herbal medicine into Ghana’s healthcare system is a significant step forward, there remains a dearth of evidence regarding satisfaction levels and the concurrent use of herbal and modern medicines in the treatment of malaria.

Previous studies have examined the implementation and effectiveness of Ghana’s integrated healthcare system, particularly in the Ashanti region. These studies have highlighted challenges such as poor regulation and ineffective policy implementation, which have hindered the full realisation of the system’s potential [19–21]. Given the endemic nature of malaria in Ghana and the ongoing efforts to control its prevalence, this study aims to contribute to the existing body of knowledge by exploring the experiences of biomedical healthcare providers (medical doctors and pharmacists) across diverse ecological zones regarding the impact of integrated healthcare practices on malaria control using Donabedian’s framework for evaluating quality of healthcare as a philosophical bedrock.

### Theoretical framework

The Donabedian’s framework for evaluating healthcare quality provided the philosophical foundation for this research. This framework encompasses three key elements: structure, process, and outcome [26]. The ‘structure’ element refers to the fundamental physical and tangible aspects of healthcare service provision, including the availability of integrated hospitals, personnel, medications, and equipment. In contrast, ‘process’ pertains to the delivery of healthcare services and associated workflows, such as referral systems and cooperation among healthcare providers. Finally, ‘outcome’ reflects the effects of healthcare on a specific population, with positive outcomes representing the desired results [27]. The Donabedian framework was selected for its capacity to identify structural and process factors that may facilitate or hinder the practice of integrated care and malaria control.

## Methods

### Research design

This research employed phenomenological design [28] to explore the experiences of orthodox medicine providers regarding the integration of herbal medicines into the mainstream healthcare system for managing malaria cases in Ghana. Phenomenology allows researchers to investigate the lived experiences of individuals in relation to specific phenomena or events [28]. By focusing on the shared experiences of a study population, phenomenology aims to distil individual accounts into a collective or universal essence [28].

### Study setting

The study was conducted in Ghana, where malaria remains one of the leading causes of morbidity and mortality, disproportionately affecting various demographic and socio-economic groups [29]. The capitals of the Ashanti, Central and Upper West regions were selected as specific study sites due to their high malaria prevalence rates—15–20% in Ashanti, 20–25% in Upper West, and 25–30% in Central regions [30].

In addition, integrated hospitals and numerous privately owned herbal medicine clinics are located in these regional capitals, making them suitable sites for the study. For example, the Kumasi metropolis hosts three integrated hospitals (Kumasi South Hospital, Suntreso Government Hospital, and Tafo Government Hospital), while Cape Coast has two (Cape Coast Metropolitan Hospital and Ewim Polyclinic), and Wa municipality includes one (Wa Municipal Hospital) [31]. The presence of these healthcare facilities underscores the necessity of exploring the role of integrated healthcare practices in managing malaria cases.

### Participants

The study participants included medical doctors (MDs) and pharmacists (PMs). Given the focus on integration and malaria control, these orthodox medicine providers were deemed eligible as their experiences could significantly inform policy development and amendments aimed at enhancing evidence-based practices, improving the integration of herbal medicine, and ultimately reducing malaria prevalence. Participant selection was primarily guided by specific criteria, such as age, type of facility (integrated hospital) and geographic location. Thus, the study population consisted of experienced medical doctors and pharmacists aged 18 or older who were practicing in integrated hospitals in the Kumasi, Cape Coast metropolis and Wa municipality. Participants were required to be at least 18 years of age to ensure their ability to offer informed consent, consistent with Ghana’s legal and ethical standards [32].

Community members were excluded from the study, as a recent Ghanaian investigation has already assessed the experiences of service users regarding integrated healthcare practices [19]. This research aims to extend the findings of Ampomah et al. by delving into the experiences of orthodox medicine providers in relation to integration and malaria management. Additionally, policymakers were excluded to concentrate on the experiences of grassroots participants—those directly involved in healthcare delivery whose activities are influenced by integration practices.

### Sampling technique

A purposive sampling technique was employed to select participants from six integrated hospitals across three study sites: Kumasi [3], Cape Coast [2], and Wa [1]. Participants were chosen based on their demonstrated knowledge of the research topic, their motivation to participate, and their willingness to engage in the study. Potential participants were contacted individually to provide detailed explanations of the study’s purpose and objectives. Following approval from the management of each hospital, participants who voluntarily provided informed consent were recruited and interviewed. This purposive sampling approach enabled the selection of participants who were well-positioned to provide rich and insightful data relevant to the research question.

### Data collection instrument and procedure

Data were gathered using a semi-structured interview guide, designed in accordance with the Donabedian framework for assessing healthcare quality (see Supplementary file 1). The same interview guide was employed for both groups and included questions that explored participants’ knowledge regarding the role of integration in malaria control, challenges associated with the practice of integration, and its outcomes in managing malaria in Ghana.

Fieldwork for data collection was conducted from July to October 2023, with face-to-face, in-depth individual interviews taking place in participants’ offices at their preferred times. Three research assistants (two males and one female) were involved in the data collection process, with one assistant assigned to each setting. Two assistants were recruited from the University of Cape Coast (UCC), while the third was from the University of Development Studies (UDS), Wa campus. All assistants held master’s degrees in public health and were well-versed in qualitative research. They received training using an information sheet and the interview guide to ensure a thorough understanding of the study objectives. Reflexive field notes were maintained throughout the data collection period. The interviews, conducted in English, were audio recorded and lasted, on average, 50 min. The first author (IGA) was present during the first three interviews to ensure accuracy in the interview process. Generally, participants were asked to share their views regarding the role herbal medicine plays in healthcare delivery in Ghana. This was followed by the question ‘Are you aware of the practice of integrated healthcare (herbal medicine integration) in Ghana?’ (see Supplementary file 1). Probing was used to solicit detailed responses. Prior to the actual data collection, the interview guide was pre-tested with two pharmacists and one medical doctor. The pretesting exercise helped in modifying the instrument to gather meaningful and reliable data. Data saturation guided the sample size, with recruitment ending at 26 participants when no additional information or themes emerged from the data.

### Data analysis and rigour

A professional transcriber transcribed all 26 interviews. IGA reviewed the recordings and compared them with the transcriptions to ensure data accuracy. Initial transcripts were reflected upon and coded before subsequent interviews were conducted. Although reiteration of the interviews was deemed unnecessary, clarifications were sought from some participants post-data collection.

Data analysis was conducted using NVivo version 12 software (QSR International Pty Ltd, Victoria, Australia) [33] and a framework analytical approach. This procedure involves both inductive and deductive analytical processes, comprising five steps: familiarisation, defining a thematic framework, indexing, charting, and mapping and interpretation [34].

Two authors (IGA and TIE) conducted data analysis independently. The transcribed data were meticulously reviewed multiple times to ensure familiarity with the content. Following this initial examination, a thematic framework was established based on notes taken during the familiarisation process. Key ideas articulated by the participants were identified inductively at this stage. The subsequent step involved indexing, during which sections of the data were marked according to distinct key concepts or themes. An inductive approach was also employed during this phase to ensure that the themes emerged directly from the data. Following indexing, charting was performed, categorising the marked data into charts aligned with the identified themes. Finally, mapping and interpretation were undertaken, arranging the charted data to illustrate participants’ experiences regarding the integration of herbal medicine into the mainstream healthcare system for malaria control in Ghana. During the mapping and interpretation stage, a deductive analytical technique was utilised to group the themes according to the elements of Donabedian’s framework for evaluating healthcare quality. Further exploration of themes showed direct linkages with the components (structure, process, and outcome) of the framework. Themes that linked to infrastructure and direct service provision were categorised under the structure and process components, respectively. Conversely, the consequences or impacts of integrated healthcare practices on malaria control were discussed within the outcome component. For instance, the availability of integrated hospitals was classified under the structure component due to its association with infrastructure. Similarly, follow-up services, being a health service delivered within the system was linked to the process component. The second author, GAA, cross-checked the emergent themes to confirm their alignment with the participants’ narratives. The themes are presented alongside illustrative quotations, each followed by the respective participant’s details (e.g., PM 1, Cape Coast). The Consolidated Criteria for Reporting Qualitative Studies (COREQ) checklist [35] was employed to guide and review the final manuscript (see Supplementary file 2, COREQ Checklist).

Several strategies were used to further strengthen the research’s credibility/trustworthiness, including:

- Researcher/assistant triangulation: Uniformity in data collection and interpretation was maintained through discussions between the lead author (IGA) and research assistants.
- Member checking: Sharing transcribed interviews with some participants for authentication and potential amendments allowed for participant validation of the interpretations.
- Thorough description of study setting and methods: Detailed information regarding the study setting and methodological approach offers transparency and facilitates replicability [36, 37].

## Results

### Background characteristics of participants

The study involved 26 participants, comprising 11 medical doctors and 15 pharmacists. The proportion of female medical doctors (54.5%) was slightly higher than that of their male counterparts. In contrast, a reverse trend was observed among pharmacists, with a slight majority of male participants (53.3%). The ages of the medical doctors ranged from 25 to 39 years, with a mean age of 32 years, while pharmacists’ ages varied from 23 to 53 years, with an average age of 35.3 years. The majority of medical doctors hailed from the Kumasi metropolis (5), with three participants each from the Cape Coast metropolis and Wa municipality. Among the pharmacists, seven were from the Wa municipality, five from Kumasi and three from the Cape Coast metropolis. Most participants (50%) identified as Akans, while other ethnic groups comprised 23.1%, Ewe (11.5%), Waala (11.5%), and Ga Adamgbe (3.9%) (see Table 1).

#### From

Integrating modern and herbal medicines in controlling malaria: experiences of orthodox healthcare providers in Ghana.

**Table 1:** Characteristics of study participants in Cape Coast, Kumasi and Wa, July - October 2023 (*N* = 26)

Participants’ characteristics	Frequency (***n***)	Percentage (%)
**Profession**		
Medical doctors	11	42.3
Pharmacists	15	57.7
**Sex**		
*Medical doctors*		
Males	5	45.4
Females	6	54.5
*Pharmacists*		
Male	8	53.3
Female	7	46.7
**Place of operation**		
*Medical doctors*		
Coastal (Cape Coast)	3	27.3
Forest (Kumasi)	5	45.4
Savannah (Wa)	3	27.3
*Pharmacists*		
Coastal (Cape Coast)	3	20
Forest (Kumasi)	5	33.3
Savannah (Wa)	7	46.7
**Ethnicity**		
Akans	13	50
Waala	3	11.5
Ewe	3	11.5
Ga-Adamgbe	1	3.9
Other	6	23.1

### Emergent themes

The framework analysis yielded five principal themes: effectiveness, knowledge about the practice of integration, structures related to integrated health service delivery, processes involved in the provision of integrated healthcare, and outcomes/effects of integrated care practices on malaria control (see Table 2).

#### From

Integrating modern and herbal medicines in controlling malaria: experiences of orthodox healthcare providers in Ghana.

**Table 2:** Themes generated from accounts/experiences of participants in Cape Coast, Kumasi and Wa, July – October 2023

Main theme	Sub-theme
Effectiveness of herbal medicine	
Knowledge about the practice of integrated healthcare	
Structures associated with integrated health service delivery	Availability of integrated hospitals Inadequate/scarcity of medical herbalists Insufficient equipment and medications
Processes involved in the provision of integrated care	Follow-up Hospital-based challenges • Disapproval of orthodox healthcare providers to herbal medicine usage • Uncoordinated health service delivery • Nature referral structure National/policy related challenges • High cost of herbal medicine resulting from limited NHIS • Inadequate advertising exercises on the practice of integration • Inadequate education/training on herbal medicine • Unstandardised herbal medicine practice due to poor regulation
Outcome/effect of integrated care on malaria control	

As illustrated in Table 2, not all primary themes were further subdivided into subthemes. For example, while ‘structure and processes involved in the provision of integrated care’ was a primary theme with corresponding subthemes, ‘outcome/effect of integrated care on malaria control’ was not. Table 3 provides a detailed breakdown of the ‘process’ primary theme, including its associated subthemes and codes.

#### From

Integrating modern and herbal medicines in controlling malaria: experiences of orthodox healthcare providers in Ghana.

**Table 3:** An illustration of process as a main theme with its corresponding subthemes and codes - analysis of reports from participants in Cape Coast, Kumasi and Wa, July – October 2023

Main theme	Subtheme	Codes	Category
Processes involved in the provision of integrated healthcare	Hospital-based challenges	1. Disapproval of orthodox healthcare providers to herbal medicine usage 2. Uncoordinated health service delivery 3. Nature referral structure	Barriers hindering the effect of integrated healthcare on malaria control
	National/policy related challenges	1. High cost of herbal medicine resulting from limited NHIS 2. Inadequate advertising exercises on the practice of integration 3. Inadequate education/training on herbal medicine 4. Unstandardised herbal medicine practice due to poor regulation	Barriers hindering the effect of integrated healthcare on malaria control
	Follow up	Follow up	Enabling factor enhancing the effect of integrated healthcare on malaria control

#### Effectiveness of herbal medicine

Some pharmacists indicated that herbal medicine plays a beneficial role in the Ghanaian health system, citing positive treatment outcomes. They noted that herbal remedies serve as first point of call or substitutes/alternatives to modern medicine, effectively managing conditions such as malaria, snake bites, tuberculosis, and hepatitis. Some pharmacists remarked:

*They play a positive role because in actual fact*,* their outcomes are good. They can actually be used in place of our orthodox medicine to manage conditions. And as I said*,* I have seen many times where herbal medicine helped in the management of common diseases like malaria*,* tuberculosis*,* hepatitis*,* etc. Then they have been used in managing feverish conditions* [PM 10, Kumasi].

*I think that usually herbal medicines are more of the first call of contact*,* especially in our homes. Here in the Upper West region*,* when you take patients’ history*,* you will see that they have already used some herbal medications before coming to the hospital. So*,* it is helping the community. For example*,* the practitioners offer anti-snake bite medications to the people* [PM 15, Wa].

*…. what I have realised is that clients who have had access to herbal medications have given positive feedback. They got what they intended to achieve in terms of their wellbeing. So*,* I think the herbal field is playing a significant role in our health system because people are seeing results* [PM 1, Cape Coast].

#### Knowledge about the practice of integration

Participants demonstrated varying levels of awareness regarding the practice of integration in Ghana. Medical doctors were generally knowledgeable about the establishment of a herbal medicine department at KNUST and the creation of herbal clinics in selected health facilities. However, some medical doctors were unaware that the malaria treatment protocol had been amended to include the recommendation of herbal anti-malaria drugs. Conversely, pharmacists were well-informed about the recent amendment to Ghana’s anti-malaria policy, which now includes FDA-approved herbal medications in treatment plans. One medical doctor noted:

*…there is a full course at KNUST that trains physician assistants in TM. Then, there are some few healthcare facilities that have the integrated system* [MD 9, Kumasi].

Another commented:

*I know that there is a herbal unit here in our hospital but I didn’t know that it has been integrated for the treatment of malaria such that if artemether lumefantrine is not working or if the patient prefers TM*,* then that option should be made available to them* [MD 7, Kumasi].

A pharmacist added:

*…there has been an update on our anti-malaria policy. Going through the section on treatment on malaria*,* there is a section that talks about malaria treatment with herbal medicine. It says that malaria treatment with herbal medicine will be by the recommended anti-malaria herbal medicine approved by the FDA. This information was not in the past document*,* but it is now in the amended policy document. So*,* any anti-malaria that has been approved by the FDA can be considered* [PM 13, Wa].

### Structures associated with integrated health service delivery

The theme of structure encompasses the physical environments in which integrated health services are provided. Several sub-themes emerged under this overarching theme, including the availability of integrated hospitals, the scarcity of certified herbal medicine providers, and shortages of equipment and medications.

#### Availability of integrated hospitals

Participants indicated that there is a limited number of integrated hospitals in Ghana. Respondents from the Wa municipality noted that only the Wa Municipal Hospital functions as an integrated facility. Similarly, participants from the coastal region expressed concerns regarding the insufficient number of health facilities offering integrated services, leading to perceptions that such services are not readily accessible to those seeking care. A medical doctor from Kumasi remarked:

*In Kumasi here*,* I know of Tafo government hospital and Atonsu (Kumasi South hospital) that have the herbal units* [MD 5, Kumasi].

Another participant from Wa confirmed:

*So*,* far*,* it is only the Wa Municipal hospital that is an integrated health facility in the Wa municipality* [PM 6, Wa].

A doctor from Cape Coast added:

*Aside my facility*,* I know LEKMA has a herbal unit integrated into their system. That is the only other place that I have worked. Seeing that I know of only one facility besides my current facility*,* I will not say that it [integrated healthcare service] is readily available to patients* [MD 4, Cape Coast].

#### Inadequate/scarcity of medical herbalists

Participants, regardless of their professional background or location, expressed concerns regarding the insufficient number of qualified medical herbalists. It was reported that typically only one medical herbalist is assigned to each facility, often supported by a nurse. One participant noted:

*The number of medical herbalists is few. In our facility*,* we have only one person. So*,* that should be increased* [Participant 7, MD, Kumasi].

Another participant mentioned:

*The medical herbalist is normally given a nurse to assist him with the clients. The practitioners at the unit are not many* [PM 4, Wa].

A participant reflecting on staffing challenges stated:

*I don’t know whether to call it inadequate staff*,* or failure to absorb the herbal practitioners. I can’t phantom why for the past years*,* we didn’t have this. But in my view*,* I will say that there weren’t enough staff to support this (integration)* [PM 14, Wa].

#### Insufficient equipment and medications

Participants frequently raised issues regarding the insufficiency of medications and the lack of adequate working space as significant barriers to effective integration and malaria control. This sentiment was particularly prevalent among respondents from the coastal region, who noted that patients suffering from malaria often avoid seeking treatment at herbal units due to the inconsistent availability of medications, resulting in interrupted treatment courses. One medical doctor stated:

*…they don’t have most of the medications. So*,* in most cases*,* they [patients] don’t go there for malaria treatment*,* except for the chronic conditions that they have the stock of medication. Even with that*,* assuming they go for the medication today*,* they will not get another stock for a period of time. So*,* treatment is truncated at a point* [MD 3, Cape Coast].

Another doctor elaborated on spatial constraints:

*When you come to the facility*,* we lack space for a whole lot of other units. So*,* you can see that where we are supposed to do our diagnostics is where we have merged with the TM unit. I don’t know for the other healthcare facilities*,* but I can talk for my facility. There is not enough space for them to practice* [MD 1, Cape Coast].

A pharmacist commented on the shared operational equipment:

*I don’t think they have anything different from what we use. From what I have seen with the person I am working with; if it is malaria*,* he will let the patient go for a lab test. Then*,* based on that result he will give the herbal medication. So*,* they use similar equipment like we do* [PM 2, Wa].

### Process involved in the provision of integrated care

This theme addresses the workflows associated with delivering healthcare services to patients. Key ideas that emerged were categorised into three sub-themes: follow-up, hospital-based challenges, and national/policy-related challenges.

#### Follow-up

Participants provided insights into the importance of follow-up activities in maintaining client relationships and ensuring patients are responding to treatment. One medical doctor noted:

*In fact*,* the herbal practitioner that I work with does that. When he gives herbal medicine to patients*,* he follows up to make sure the patient is doing well* [MD 2, Wa].

Another participant expressed satisfaction with their herbal unit’s follow-up practices:

*For our facility*,* I think our herbal unit is doing fantastic even though we are not giving them enough support. They are doing fantastically well. They are able to maintain their clients through follow ups*,* tracing* [PM 8, Cape Coast].

#### Hospital-based challenges

This section delineates the hospital-level challenges that hinder the effective management of malaria through the integration of herbal medicine into orthodox practices. The primary obstacles identified include the reluctance of orthodox healthcare providers to accept herbal medicine, the lack of coordinated health service delivery, and the nature of the referral structure. Participants reported that some medical doctors harbour negative perceptions regarding herbal medicines, resulting in a general disapproval of their use. This disapproval creates barriers to collaboration with medical herbalists, even in situations where treatment has failed.

One participant remarked,

*…. the perception that herbal medicine is not good by those of us in the mainstream is an issue. So*,* there were situations where they (medical doctors) have to direct patients or collaborate with the medical herbalist during treatment failure but that did not happen because they disapprove of herbal medicines* [PM 10, Kumasi].

Medical doctors expressed their dissatisfaction with the integration intervention, asserting that the mere presence of a herbal clinic within the hospital does not ensure coordinated service delivery. According to them, medical herbalists do not contribute to the malaria treatment plan due to the lack of collaboration between orthodox and herbal healthcare providers. One doctor stated:

*…in our practice*,* when we get patients and we are managing malaria*,* we do not consult the medical herbalists. And if we are really integrated*,* when patients are here*,* why don’t they [medical herbalist] come around and give their contribution that they have medications to help the patient. But in our case*,* it is only we the clinical practitioners who come around; you don’t see the medical herbalists. So*,* I will not say that we are fully coordinated. They have a unit here*,* but we are not integrated* [MD 7, Kumasi].

The issue of uncoordinated health service delivery is closely linked to the nature of the referral structure within Ghana’s integrated healthcare system. Providers from the Wa municipality and Cape Coast metropolis indicated that the decision to refer patients to medical herbalists is contingent upon the complexity of the malaria case and the patient’s preference. Some medical doctors are open to referring uncomplicated malaria cases to herbal practitioners, while others leave the decision to the patient, believing that the orthodox health system possesses the requisite medications for treating malaria. Pharmacists corroborated this assertion, noting that most referrals from medical doctors to herbal clinics tend to be for chronic conditions rather than malaria.

One medical doctor explained,

*…if I come into contact with these patients*,* especially the severe malaria*,* I will not call the herbal person because he is going to waste my time. Once the person has gone into severe malaria and especially speaking from the children’s perspective*,* kidneys are going to shut down and the liver. So*,* it is now affecting the organs and not just a normal malaria. So*,* I am taking care of the liver while managing the malaria. So*,* I don’t think I will call the herbal person. But for the uncomplicated malaria*,* I don’t have an issue with that*,* I refer* [MD 2, Wa].

Another physician added,

*I don’t think any orthodox practitioner has ever referred a patient to the herbal unit. We leave that to the patient to decide. If we establish the fact that the person has malaria*,* we have all the necessary orthodox medicines to treat malaria. But if the patient is an adult and prefers herbal medicine*,* we give them the opportunity to decide. But for us to refer to herbal practitioners*,* no!* [MD1, Cape Coast].

A participant from a herbal clinic remarked,

*…for this facility*,* we do have referrals. But I have not seen them (medical doctors) do referrals for malaria cases. It is the chronic conditions that I have seen them do a number of referrals to the herbal unit. But for malaria*,* I haven’t seen them do such referrals* [PM 7, Cape Coast].

#### National/policy related challenges

This theme highlights the national-level challenges and policy-related issues that impede effective malaria control through the integration of herbal medicine. The primary challenges identified include the high cost of herbal medicine due to limited coverage by the National Health Insurance Scheme (NHIS), inadequate promotional activities regarding integration practices, insufficient education and training on herbal medicine, and the lack of standardised practices resulting from poor regulation. Participants noted that orthodox malaria treatments are covered by the NHIS, making them financially accessible for patients; in stark contrast, herbal medicine clinics do not benefit from such coverage. This disparity, particularly emphasised by medical doctors in the Kumasi metropolis, suggests that the limited scope of health insurance is a significant barrier to reducing malaria prevalence in Ghana.

One doctor stated,

*Orthodox medications for malaria treatment are very cheap but I don’t know about herbal medicine because that is not my field. The NHIS covers the orthodox medication but not for herbal medicine* [MD 7, Kumasi].

Moreover, participants highlighted that the lack of adequate promotional activities regarding the integration of herbal medicine poses a significant challenge to effective malaria control in Ghana. They argued that insufficient publicity has resulted in a knowledge gap among both healthcare providers and service users. A doctor from remarked:

*I believe that it is not well-known that there is something like herbal medicine integration. So*,* publicity is a challenge. I didn’t know about the medical herbalist in the hospital until recently. Meanwhile*,* I have been working with them. They will be in their consulting room*,* and I will be in my consulting room. But I wasn’t aware that there was an integration* [MD 2, Wa].

A pharmacist elaborated:

*The main problem is that many people don’t know that TM is being used in the hospital to treat both the clients and the general public. They don’t know that herbal medicine has been improved in terms of professionalism* [PM 2, Kumasi].

Another doctor added:

*From the adverts I have been seeing on TV*,* I don’t think people are aware of the TM integration system. I don’t think even the people producing these herbal medications are aware of this. It is always like it is either you choose orthodox or herbal medicine. So*,* I am not sure people know about the integration* [MD 4, Cape Coast].

The need for formal education and training for orthodox medicine providers emerged as a crucial factor in bridging the gap between orthodox and herbal healthcare practitioners. Participants noted that while both groups expressed a willingness to engage in training on herbal medicines, such opportunities were rarely presented. Medical doctors reported that their interactions with medical herbalists typically occurred in practice settings, a context that they found challenging, as it limited their understanding and integration of herbal practices into their treatment approaches. They emphasised that education and training would enhance their exposure to herbal medicines, thereby broadening their knowledge base and improving patient care within the integration programme.

One participant stated,

*…we don’t have that much exposure. I feel like we don’t really have a lot of education being done for us who do not do herbal medicine. We have a background in pharmacognosy*,* but I feel like that is where it ends and that is not helpful*.

[PM 12, Kumasi].

Another commented,

*Unlike the pharmacy school where they get the opportunity to sit in the same class with herbal practitioners*,* we go through medical school without having that experience or exposure. So*,* you only come to meet the person in the field. So*,* it is like you are now trying to understand the person and incorporate their ideas into your practice. That is a bit challenging* [MD 10, Wa].

A third doctor expressed concern regarding the lack of training opportunities:

*…with regards to the training period*,* there is no time or is there a space for TM training. And since I started working*,* I haven’t had any workshop or training on that. The pharmaceutical companies try to advertise herbal medicine. They don’t really provide training. I have not been involved in any formal training on herbal medicine but acquiring such training would help broaden my knowledge in the field of herbal medicines and help in offering patients the best services* [MD 4, Cape Coast].

Discussions regarding the registration, regulation, and standardisation of herbal medicine practice revealed that many orthodox healthcare providers perceive the field of herbal medicine as lacking adequate standardisation due to insufficient regulations. Participants noted that orthodox medicine producers adhere to established standards, providing comprehensive information on dosage that mitigates the risk of liver and kidney damage. In contrast, they expressed concerns that similar standards do not apply within the herbal medicine sector. Medical doctors highlighted that effective regulation enables manufacturers of orthodox medicines to recall faulty production batches, a process they believe is absent in herbal medicine practice. Pharmacists from the forest and savannah zones emphasised that inadequate information regarding herbal medicine, particularly concerning dosage, hampers the integration’s potential to contribute to malaria control.

One participant articulated a common concern:

*Let me not just speak from the point of view of malaria but in general. Personally*,* when it comes to herbal medicine*,* my problem with it is the standardisation and understanding the implications it has on your organs like the kidney and liver. Because you will realise that a lot of times*,* the damage is to the liver and kidney because that field of medicine is not properly regulated*,* so people act anyhow* [PM 12, Kumasi].

Another participant raised concerns about dosage clarity:

*… when you give me herbal preparation and then you tell me to take one cup*,* I don’t know how much my body needs for a day or per hour. You have not told me how much of the ingredient is in one cup. So*,* what if whatever that is in there is too strong for my liver to metabolise? Yes*,* all malaria medicines come from the same plant whether herbal or orthodox. So*,* if orthodox has been able to tell me that there is this milligram in this dose*,* but you are not able to tell me*,* then at the end of the day*,* it means I have put my liver and kidney at stake. I think that is one of the reasons why we have a lot of kidney and liver problems* [PM 15, Wa].

Another medical doctor added,

*…from the beginning to the end*,* you don’t really know how it is being done. If you go to Kinapharma*,* for instance*,* they are doing things in a particular standard. There is a quality assurance unit. So*,* if there is an issue with a particular batch*,* that batch can be recalled. For the herbal*,* there may be very few people who do things by proper standards. I know there is a herbal medicine regulatory body but I don’t know how their things are done. And so*,* you cannot be certain. The rooter mixture that I buy; will it be the same as what I will buy tomorrow?* [MD 4, Cape Coast].

### Outcome/effect of integrated care on malaria control

The integration of herbal medicine into conventional healthcare practices in Ghana has elicited mixed responses from medical professionals regarding its efficacy in malaria control. Many physicians, particularly those based in coastal and savannah regions, expressed skepticism about the effectiveness of integrated healthcare in combating malaria. These practitioners argued that the lack of adequate resources—specifically, insufficient herbal anti-malarial medications and necessary medical equipment—has severely limited the potential benefits of such integration. As a result, they noted, patients often resort to purchasing herbal malaria treatments from informal street vendors, which can lead to adverse health outcomes.

One physician articulated this concern, stating,

*The reason why I am saying it hasn’t helped is that even the facilities where the integration is going on are understocked. So*,* assuming you even refer a patient there*,* they do not have medications for them. If you like I will send you to Ewim’s TM unit. There are no drugs there – same for metro. So*,* it is like the normal Ghanaian talk. We have created a herbal unit. The person is there but they don’t have the things to work with. And so*,* patients end up buying the herbal malaria medications that we don’t want them to buy on the street and complications occur* [MD 3, Cape Coast].

Another doctor echoed similar sentiments, emphasising that herbal practitioners often default to prescribing standard anti-malarial medications due to a lack of traditional alternatives:

*I don’t think the herbal practitioner in the facility has stock of traditional anti-malarial drug. So*,* I would say the integration has not helped because even the herbal medicine practitioner prescribes the normal anti-malaria medications instead of the herbal ones* [MD 10, Wa].

Furthermore, one physician reflected on the broader context of malaria interventions, stating,

*…. we have malaria intervention such as the treated nets*,* residual spraying*,* seasonal prophylaxis*,* roll-back malaria campaign and so on*,* I am just giving you this background. All these were done to control malaria. It has been long overdue especially in regions like these where the statistics is not looking good for malaria. So*,* when you look at all these interventions*,* the herbal integration is missing. So*,* I don’t see where they are. That is why I am saying that they are doing their bit*,* but on the bigger picture*,* we are not seeing the contribution of the integration* [MD 2, Wa].

In contrast to the views of medical doctors, pharmacists, particularly those situated in forested areas, presented a more optimistic perspective on the integration of herbal clinics within hospitals. They argued that these clinics provide an alternative approach to malaria treatment that is both effective and beneficial. These pharmacists noted that the integration has facilitated access to approved herbal anti-malarial medications, offering patients viable alternatives when conventional treatments are unavailable. One pharmacist stated,

*It has helped in creating alternative medicines for malaria control. With this integration*,* we now know that we have approved herbal medications that we can use to get the desired outcomes. So*,* even if we find ourselves in situations where the orthodox medicines are not available*,* we do fall on the herbal medicines. Like I mentioned about the antimicrobial resistance*,* we need to have more alternative medicines so that the organisms do not become resistant to the orthodox medicines* [PM 10, Kumasi].

This dichotomy in perspectives highlights the complexities surrounding the integration of herbal medicine into malaria control strategies in Ghana, calling for further investigation into resource allocation and the potential for collaboration between conventional and herbal practitioners to enhance malaria management outcomes.

## Discussion

Ghana’s determination to prioritising herbal medicine within its health system is exemplified by the establishment of herbal medicine clinics in select government-owned hospitals [38, 39]. This research provides valuable insights into the contributions of integrating modern and herbal medicines for malaria control, employing the Donabedian framework for evaluating healthcare quality.

### Structural considerations

The integration of herbal clinics into public hospitals in Ghana has created a promising structural framework for promoting the safety and efficacy of herbal treatments for malaria [20]. However, the study findings indicate that access to integrated health services is significantly hindered by inadequate facilities, equipment, and medications. Earlier studies have echoed similar findings [20, 21, 40]. To optimise the impact of this integration, policymakers must prioritise increasing the number of integrated hospitals, ensuring a consistent supply of certified herbal anti-malarial medications, and expanding the workforce of qualified medical herbalists within these facilities.

### Process considerations

The study’s findings align with previous research, which has explored the dynamics of referrals between herbal and orthodox medicine providers [19–21, 41–44]. While referrals are influenced by factors such as malaria severity and patient preference, they suggest a level of collaboration at the health facility level. Evidently, a well-organised referral system could greatly benefit the Ghanaian health system in managing malaria [45]. Establishing efficient inter and intra referral networks may serve as a viable integrative strategy to reduce malaria prevalence or potentially eradicate the disease [21, 38, 45], making it an imperative issue for policy makers to consider in health policy development.

Many countries have successfully integrated traditional and herbal medicine into their national healthcare frameworks. For instance, traditional medications are utilised at all levels of healthcare in China, covered by both public and private insurance [46, 47]. Similarly, herbal medicine practitioners are permitted to operate in both public and private hospitals in South Korea and Vietnam, with their services also covered by insurance. In Switzerland, five complementary therapies are included in the mandatory health insurance programme, provided that the prescribing practitioner holds a certification in complementary and alternative medicine [48]. In Ghana, studies have shown that the National Health Insurance Scheme (NHIS), the country’s main health financing policy have enhanced health service utilisation [49–51]. However, the NHIS was not designed to accommodate integration programmes. The absence of herbal anti-malarial medications on the NHIS essential medicines list poses a significant barrier to integration and malaria control, as patients seeking treatment from herbal units in integrated hospitals are required to pay out-of-pocket for their medications [21]. To further enhance the integration of herbal medicine into malaria management, it is imperative to include safe and effective herbal remedies on the essential medicine list. This recommendation is consistent with findings from other studies [21, 52].

The importance of advocacy in promoting effective malaria control through integration is underscored by this study. It appears that public awareness in Ghana regarding the incorporation of herbal medicine into the mainstream healthcare system specifically, the availability of herbal anti-malarial services and medications has been limited by inadequate promotional activities [19]. The country’s herbal medicine policy allows media personnel to receive training to educate the public about the benefits and risks associated with herbal medicines [25]. Therefore, media outlets (including audio, audiovisual, and print) should actively promote the effective implementation of integration programmes and highlight their potential to reduce or eradicate malaria in Ghana.

In accordance with World Health Organization guidelines, health training programmes should incorporate a module on herbal medicine for students [39]. Previous studies have echoed this recommendation [19, 20], which is already being implemented in Nigeria [1]. In Ghana, a herbal medicine department has been established within the faculty of pharmacy at KNUST to train qualified medical herbalists; however, the country has yet to fully adopt and implement the WHO’s recommendation to expand herbal medicine training. Acquiring comprehensive knowledge on herbal medicine is crucial for orthodox healthcare providers because it can enable them to understand patient preferences for herbal treatments and facilitate informed decision-making about health issues [39].

### Challenges with herbal medicine practice in Ghana

Concerns surrounding the standardisation and dosing of herbal medicines can lead to incidents of overdose, as many medicinal plants contain compounds that may be harmful if consumed inappropriately. Numerous herbs are known to contain substances such as acids, phenolic compounds [53, 54] tetrahydrocannabinol [55], serpentine [56], and various active compounds like phenols, alkaloids, saponins, and tannins [57]. This underscores the potential for herbal medicines to exacerbate health problems if proper dosing is not established and adhered to. Addressing dosage issues may be achieved by enhancing healthcare providers’ knowledge through formal training. Furthermore, herbal medicine practitioners could benefit from the sharing of knowledge and research findings related to their treatments, which could lead to the standardisation of drugs, dosages, and efficacy [58, 59].

Additionally, health regulatory agencies should rigorously test and validate the appropriate dosages and effectiveness of herbal medications before granting licenses for public use. The strict enforcement of laws and protocols governing herbal medicine practices would significantly contribute to improving the quality and safety of herbal anti-malarial treatments in Ghana.

### Outcome considerations

The integration of herbal and conventional health systems reflects the government’s commitment to improving the efficiency and scope of healthcare delivery in Ghana [47, 60]. Participants, particularly pharmacists, highlighted that integrated healthcare practices have positively impacted malaria management by preventing the development of resistance to conventional anti-malarial medications, as they can alternate between herbal and orthodox treatments. Conversely, medical doctors expressed differing views, suggesting that the impact of integration on malaria control is minimal compared to that of other malaria control initiatives. This divergence highlights the varying experiences and perspectives among orthodox healthcare providers regarding the efficacy of integration.

To enhance the significance of integration in malaria control demands the involvement of all stakeholders—including clients, healthcare providers, health system researchers, legislators, regulatory bodies, and the media—through active collaboration and engagement.

### Research implication

Public health experts, researchers in consumer engagement and herbal medicine, and social scientists should collaborate to conduct comprehensive research addressing critical questions that could bolster the contributions of the integration programme to malaria control in Ghana. For instance, employing both qualitative and quantitative methodologies may be necessary to ascertain the knowledge, attitudes, and perceptions of stakeholders, particularly hospital managers and heads of health directorates, regarding herbal therapy, its integration, and its potential benefits in combating infectious diseases in Ghana.

### Strengths and limitations

The findings of this study may not be generalisable due to its qualitative research design. Future research could adopt a mixed-methods approach to explore the understanding and involvement of a broader range of orthodox healthcare professionals concerning the integration of the two health systems and its role in managing infectious diseases such as malaria. Additionally, the use of individual face-to-face in-depth interviews as the sole data collection technique may have limited the generalisability and reliability of the findings. Despite these limitations, the study corroborates previous research while also presenting unique and original findings. The recruitment of participants from Ghana’s three ecological zones (coastal, forest, and savannah) enhances the credibility and trustworthiness of the results.

## Conclusion

This study employed a qualitative approach to investigate the experiences of medical doctors and pharmacists regarding integrated healthcare practices in the treatment and management of malaria. Identifying quality care elements is crucial for improving the efficiency of healthcare interventions; hence, the Donabedian framework for evaluating healthcare quality was utilised as the theoretical foundation.

The framework analysis indicated that participants possessed knowledge regarding the integration of herbal and orthodox medicine practices within designated healthcare facilities. A key strategy identified for building and maintaining patient clientele at herbal clinics within integrated hospitals was the implementation of effective follow-up procedures. Furthermore, pharmacists expressed the belief that the integration intervention had contributed to mitigating the emergence of resistance to modern anti-malarial medications by enabling service providers to alternate between herbal and orthodox treatment modalities.

The findings also highlighted that Ghana’s integrated healthcare system faces significant challenges at both the facility and national levels, hindering its full operational capacity. At the facility level, these challenges include biomedical providers’ disapproval of herbal remedies and a lack of coordinated service delivery. At the national level, limitations include the high cost of herbal medications due to limited NHIS coverage, inadequate promotion of integration initiatives, insufficient herbal medicine education, and a lack of standardised herbal medicine practices resulting from poor regulation. To enhance the effectiveness of the integration intervention and its impact on malaria control in Ghana requires addressing these challenges. The study recommends several policy interventions, including policy amendments to support integration, revisions to the NHIS to incorporate certified herbal anti-malarial treatments, and the expansion of herbal medicine education and training for all healthcare providers. Future research should consider employing mixed-methods approaches to investigate the perspectives of policymakers, heads of health directorates, and community members regarding the role of public health interventions—such as Community-based Health Planning and Services (CHPS), integrated healthcare, and the NHIS—in addressing health inequities in Ghana.

## Electronic supplementary material

Below is the link to the electronic supplementary material.

Supplementary Material 1

Supplementary Material 2

## Data Availability

No datasets were generated or analysed during the current study.

## Abbreviations

COREQ: Consolidated Criteria for Reporting Qualitative studies
CSRPM: Centre for Scientific Research into Plant Medicine
GHP: Ghana Herbal Pharmacopoeia
KNUST: Kwame Nkrumah University of Science and Technology
MD: Medical Doctor
MoH: Ministry of Health
NHIS: National Health Insurance Scheme
PM: Pharmacist
TMPC: Traditional Medicine Practice Council
UCC: University of Cape Coast
UDS: University of Development Studies
