# Supplementary material for: Integrating modern and herbal medicines in controlling malaria: experiences of orthodox healthcare providers in Ghana
Source: Arch Public Health. 2024 Dec 23;82:240. doi: 10.1186/s13690-024-01472-5 (PMC11665210; doi:10.1186/s13690-024-01472-5)
Supplement: Supplementary file 1 — Supplementary Material 1 [file 13690_2024_1472_MOESM1_ESM.docx]

**Topic:** Integrating modern and herbal medicines in controlling malaria: Experiences of orthodox healthcare providers in Ghana

**Interview Questions:**

Medical doctors and Pharmacists in Cape Coast, Kumasi and Wa, July – October 2023

**Background characteristics of participants:**

Name of Metropolis/Municipality.....................................................................................

Age.....................................................................................................................................

Sex......................................................................................................................................

Marital status…………………………………………………………………………….

Tribe/ethnic origin.............................................................................................................

Educational level................................................................................................................

Profession………………………………………………………………………………...

Specialty of participant…………………………………………………………………..

**Structure:**

1. Please, what role does herbal medicine play in healthcare delivery in Ghana?
2. Are you aware of the practice of integrated healthcare (herbal medicine integration) in Ghana?
3. As a health practitioner, can you share with me the contribution of the practice of integrated healthcare in promoting effective malaria control in Ghana?

**Process:**

1. Please, are there any challenges associated with the practice of integration and the control of malaria in Ghana?

**Outcome:**

1. In your opinion, has the practice of integrated healthcare helped in controlling malaria in Ghana?
2. Please, in what ways do you think Ghana as a country could achieve effective malaria control through the practice of herbal medicine integration?
3. Do you have additional information regarding the topic that you would like to share with me?

**Thank you for your time and contributions.**
